# Supplementary material for: PRAME Promotes Cervical Cancer Proliferation and Migration via Wnt/β-Catenin Pathway Regulation
Source: Cancers (Basel). 2023 Mar 16;15(6):1801. doi: 10.3390/cancers15061801 (PMC10046627; doi:10.3390/cancers15061801)
Supplement: Supplementary file 1 [file cancers-15-01801-s001.zip › cancers-2287862-SI.pdf]

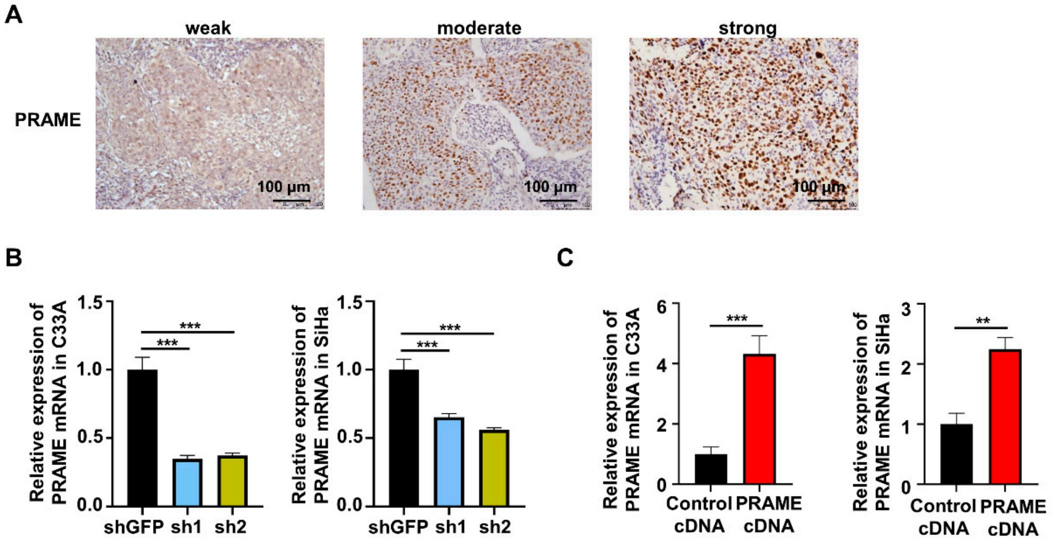

Figure S1 The images of immunostaining, and qRT-PCR confirmation of PRAME -knockdown and -overexpressed cells. (A) The example images of weak, moderate, and strong IHC staining of PRAME (1:200) (B) The transfection efficiency of PRAME knockdown in C33A and SiHa cells were verified by qRT-PCR. (C) The transfection efficiency of PRAME overexpressed in C33A and SiHa cells were verified by qRT-PCR. ** p <0.01, *** p < 0.001.
